# Diagnostic value of procalcitonin in patients with periprosthetic joint infection: a diagnostic meta-analysis

**DOI:** 10.3389/fsurg.2024.1211325

**Published:** 2024-04-10

**Authors:** Xiaobo Sun, Yijin Li, Yan Lv, Yuting Liu, Zhiwei Lai, Yirong Zeng, Haitao Zhang

**Affiliations:** ^1^ Department of Orthopaedics, Ganzhou Hospital of Traditional Chinese Medicine, Ganzhou, Jiangxi, China; ^2^ The First Clinical Medical School, Guangzhou University of Chinese Medicine, Guangzhou, Guangdong, China; ^3^ Department of Cardiology, The First Affiliated Hospital of Nanchang University, Nanchang, China; ^4^ Academic Affairs Office, Gannan Medical University, Ganzhou, Jiangxi, China; ^5^ Department of Orthopaedics, The First Affiliated Hospital of Guangzhou University of Chinese Medicine, Guangzhou, Guangdong, China; ^6^ Longhua Hospital, Shanghai University of Traditional Chinese Medicine, Shanghai, China

**Keywords:** procalcitonin, periprosthetic joint infection, diagnosis, biological markers, meta-analysis

## Abstract

**Background:**

The success rate of periprosthetic joint infection (PJI) treatment is still low. Early diagnosis is the key to successful treatment. Therefore, it is necessary to find a biomarker with high sensitivity and specificity. The diagnostic value of serum procalcitonin (PCT) for PJI was systematically evaluated to provide the theoretical basis for clinical diagnosis and treatment in this study.

**Methods:**

We searched the Web of Science, Embase, Cochrane Library, and PubMed for studies that evaluated the diagnostic value of serum PCT for PJI (from the inception of each database until September 2020). Two authors independently screened the literature according to the inclusion and exclusion criteria. The quality of each selected literature was evaluated by using the Quality Assessment of Diagnostic Accuracy Studies tool (QUADAS-2) tool. RevMan 5.3 software was used for the quality evaluation. The sensitivity, specificity, positive likelihood ratio (PLR), negative likelihood ratio (NLR), and diagnostic odds ratio (DOR) were merged by using Meta-DiSc 1.4 software. The area under the curve (AUC) and Q index were calculated after the summary receiver operating characteristic (SROC) was generated. We also performed subgroup analysis.

**Results:**

A total of 621 patients were enrolled in the nine studies. The pooled sensitivity of serum PCT for PJI diagnosis was 0.441 [95% confidence interval (CI), 0.384–0.500], the pooled specificity was 0.852 (95% CI, 0.811–0.888), the pooled PLR was 2.271 (95% CI, 1.808–2.853), the pooled NLR was 0.713 (95% CI, 0.646–0.786), and the pooled DOR was 5.756 (95% CI, 3.673–9.026). The area under SROC (the pooled AUC) was 0.76 (0.72–0.79). Q index was 0.6948.

**Conclusion:**

This study showed that PCT detection of PJI had poor diagnostic accuracy. Hence, the serum PCT is not suitable as a serum marker for PJI diagnosis.

## Introduction

Periprosthetic joint infection (PJI) is one of the catastrophic complications after total joint arthroplasty (1). The incidence of PJI after total knee arthroplasty (TKA) was 0.7% (2). The total hip arthroplasty (THA) infection incidence was 1.63% within 2 years and 0.59% between 2 and 10 years (3). PJI is the second reason for the early revision of THA (4). It may result in reduced knee or hip function and reduced quality of life for patients, as well as the failure of the implant and the need for revision arthroplasty (5).

Currently, the diagnosis for PJI depends mainly on the guidelines of the American Academy of Orthopedic Surgeon (AAOS)'s guidelines the Infectious Diseases Society of America (IDSA), the Musculoskeletal Infection Society (MSIS) criteria of 2011 and 2018, the European Bone and Joint Infection Society (EBJIS) definition, a large number of biomarkers [white blood cell (WBC), C-reactive protein (CRP), erythrocyte sedimentation rate (ESR), synovial fluid], pathologic examinations and preoperative aspirations (6–10). Unfortunately, the effectiveness of WBC in diagnosing PJI is limited (11, 12). CRP and ESR present high sensitivity and low specificity. We determine the diagnosis according to intraoperative pathologic examination with the lack of typical symptoms. It is difficult to accurately diagnose PJI before surgery, and it is a research hotspot to find specific serum markers such as interleukin-6, procalcitonin (PCT), and α-defensin (13).

PCT is a protein containing 116 amino acids produced by neuroendocrine cells and parafollicular thyroid gland cells (14). Serum PCT levels in uninfected healthy people are low but significantly increase in severe bacterial and fungal infections (15). Aseptic infections such as multiple organ failure and extensive burns are common causes of elevated serum calcitonin. It has been reported that injection of bacterial endotoxin into healthy people can induce the release of systemic PCT. The PCT test has high diagnostic accuracy in identifying systemic infection (14, 16, 17). However, different scholars have drawn entirely different conclusions regarding PCT diagnosis for PJI. Therefore, we performed a systematic review and meta-analysis to evaluate the diagnostic value of PCT in PJI.

## Materials and methods

Our study was strictly performed on the basis of the Preferred Reporting Items for Systematic Reviews and Meta-Analyses (PRISMA) guidelines (18). All authors participated in formulating the literature retrieval scheme. We finished the work in an orderly fashion.

### Data and literature sources

We searched the Web of Science, Embase, Cochrane Library, and PubMed for studies that evaluated the diagnostic value of PCT for PJI (from the inception of each database until September 2020). There was no language limit. The search strategy follows the combination of subject terms and accessible terms. The search subject words and free words are as follows: “prosthesis-related infections” or “Prosthesis Related Infections” or “Infections, Prosthesis-Related” or “Prosthesis-Related Infection” or “Prosthetic joint infection” or “Prosthesis joint infection” or “PJI” stands for disease, “Procalcitonin” or “Calcitonin Precursor Polyprotein” or “Calcitonin-1” or “Calcitonin 1” or “Calcitonin Related Polypeptide Alpha” or “Pro-Calcitonin” or “PCT” represents target index. After electronic research, additional related studies in the literature were added by manual retrieval.

### Study selection

The included studies meet the following criteria: (1) Serum PCT was used to diagnose PJI; (2) The type of study is retrospective or prospective analysis; and (3) We can indirectly or directly obtain a 2 × 2 table of PCT diagnosis for PJI; (4) The source of samples for the research is clear; and (5) The reference standard for the diagnosis of PJI is described in the literature. The following studies were excluded: (1) When we could not obtain the necessary 2 × 2 table data through calculating or contacting the author; (2) When patients suffered extra-articular infection of joint; (3) When the review literature, special case reports, animal experiments, and repeated reports were missing; (4) When the detection methods for PCT have not been used in clinical examination or have been eliminated; and (5) When the patients were in a poor nutritional state. Two authors reviewed the title, abstract, and full texts independently. The third author resolved the disagreements between the first two reviewers and made a final decision.

### Data extraction and quality assessment

Two authors independently screened the literature according to the inclusion and exclusion criteria and entered the extracted data into a table. The recorded baseline data and outcome indicators were as follows: Author, year, country, study type, gender, median age, and body mass index (BMI), detection method, threshold value, gold standard, operative site, total staff, PJI number, N-PJI number, SN (sensitivity), SP (specificity), TP (true positive), FP (false positive), FN (false negative), and TN (true negative). SN, SP, TP, and FP data were built into a 2 × 2 table.

We used the Quality Assessment of Diagnostic Accuracy Studies tool (QUADAS-2) to evaluate the quality of each selected literature (19). QUADAS-2 was adopted to evaluate the literature along four significant aspects (patient selection, diagnostic test, gold standard, and loss of follow-up). We have assessed the risk of bias in the four aspects and the clinical utility of the first three aspects. RevMan 5.3 software was used for the evaluation of quality.

### Data synthesis and analysis

We combined TP, FP, FN, and TN extracted from the literature, and obtained the SN, SP, positive likelihood ratio (PLR), negative likelihood ratio (NLR), and DOR using Meta-DiSc 1.4 software. Whether it is a PLR or an NLR, the further away from 1 it is, the more meaningful it becomes. A PLR greater than 10 means that a positive test is very effective in diagnosing the disease. An NLR less than 0.1 means that a negative test is very effective in ruling out the diagnosis (20). The higher the DOR, the better the diagnostic value (21). In addition, the area under the curve (AUC) and Q index were calculated after the summary receiver operating characteristic (SROC) was generated. An AUC value (greater than 0.8) shows good diagnostic accuracy of PCT.

In diagnostic meta-analysis, both the threshold effect and the non-threshold product can cause heterogeneity. Suppose there is a negative correlation between SN and SP (or a positive correlation between SN and 1 − SP), and there is a “shoulder-arm” point distribution on the receiver operating characteristic (ROC) curve graph. In that case, it can be judged that there is a threshold effect. When the threshold effect causes heterogeneity, Spearman correlation analysis is used. When the heterogeneity is caused by the non-threshold effect, the chi-square test is used to analyze the heterogeneity among the study results, and the inconsistency index (*I*^2^) is used as the quantitative judgment for the heterogeneity size. The greater the value of *I*^2^ expressed, the greater the value of the heterogeneity, and vice versa. *I*^2^ values of 25%, 50%, and 75%, respectively, represent low, moderate, and high heterogeneity. If the heterogeneity of multiple independent studies is low, the fixed-effect model can be selected. If the heterogeneity is high, the random effect model can be selected. Meta-regression and subgroup (Study design, Threshold value, MSIS, East Asian race) analysis are conducted to investigate the source of heterogeneity. When the heterogeneity is still high after heterogeneity analysis and treatment, we use Stata 14.0 software to draw Deeks’ funnel plot and evaluate the publication bias (22).

## Results

### Identification of studies

**Figure 1:**
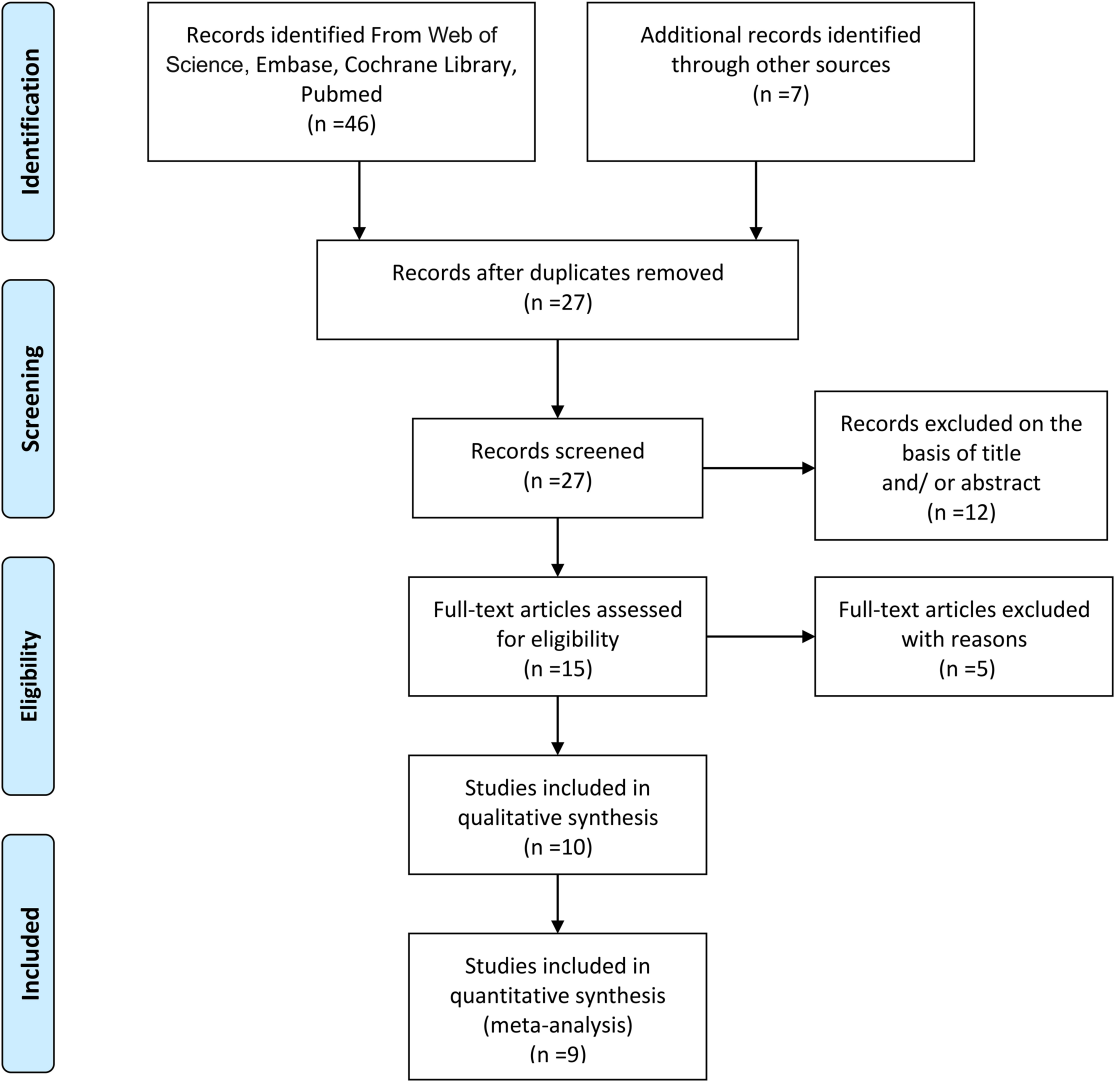
PRISMA flow diagram.

The literature screening flow diagram is detailed in Figure 1. A total of 46 references were obtained in the preliminary search in four online databases (Web of Science, Embase, Cochrane Library, PubMed). Seven additional records were identified through other sources. After removing 26 duplicates, 27 studies remained; of these, 12 articles were excluded based on the title and/or abstract, and five articles were excluded by reading their full text. One literature with qualitative synthesis was excluded. In the end, only nine studies were left for meta-analysis (14, 23–30).

### Study characteristics and patient samples

Three studies (14, 23, 26) were from East Asia and six (24, 25, 27–30) from other countries. Two retrospective studies (23, 25) and seven prospective studies (14, 24, 26–30) were included. Totally 621 patients were enrolled in the nine studies (14, 23–30), including 385 women and 236 men. The average age ranged from 62 to 72 years. BMI values were not available in four studies (23, 25, 27, 29), and those that were available in the five other studies (14, 24, 26, 28, 30) ranged from 26.7 to 30 kg/m^2^. The detection method was not found in the study (23). The threshold is 0.5 ng/ml in four studies (14, 24, 26, 29). MSIS is the “gold standard” for diagnosis in four studies (23, 25, 27, 28). All the studies included patients who underwent hip or knee arthroplasty, and only one study (24) included patients who underwent shoulder arthroplasty. The basic characteristics of the included studies are provided in Table 1. Table 2 summarizes the basic data extracted from each study (2 × 2 table).

**Table 1:** Characteristics of the studies in meta-analysis for the diagnosis of PJI applying serum procalcitonin.

Authors	Year	Country	Study type	Gender (F/M)	Median age (N/PJI)	BMI (N/PJI)	Detection method	Threshold value (ng/ml)	Gold standard^a^	Operative site
Bottner et al. (30)	2007	Germany	P	37/41	64	28.3/28	Krypto PCT	0.3	MSIS	Hip or knee
Worthington et al. (29)	2010	UK	P	25/21	72	NA.	BRAHMS PCT-Q kits	0.5	MSIS	Hip
Glehr et al. (28)	2013	Austria	P	38/46	65/66	30	Elecsys BRAHMS PCT kits	0.35	MSIS	Hip or knee
Randau et al. (27)	2014	Germany	P	73/47	67.94	NA	Immunoassay Analyzer	46	MSIS	Hip or knee
Yuan et al. (26)	2015	China	P	49/22	62/67	28.4/29.2	Immunochromatography	0.5	ICM	Hip
Yildirim et al. (25)	2017	Turkey	R	75/10	68.2	NA	The ELISA kits ab100630	0.081	MSIS	Knee
Sa-ngasoongsong et al. (14)	2018	Thailand	P	25/7	68	26.9	Enzyme immunoassay kit	0.5	ICM	Hip or knee
Busch et al. (24)	2020	Germany	P	42/28	66 /72	26.7/27.1	Enzyme immunoassays	0.5	MSIS	Hip or knee or shoulder
Chu et al. (23)	2020	China	R	21/14	65/66	NA	NA	NA	MSIS	Hip or knee

P, prospective study; R, retrospective study; NA, not applicable; N, non-PJI; Krypto PCT, time-resolved amplified cryptate emission (TRACE) technology assay; MSIS, musculoskeletal Infection Society; ICM, international consensus on infection.

^a^ Gold standard refers to the diagnostic PJI reference standard, which under the current study was considered to be 100% diagnostic accuracy.

**Table 2:** Data extracted for the construction of 2 × 2 table.

Authors	Year	Total participants	PJI	N-PJI	SN (%)	SP (%)	TP	FP	FN	TN
Bottner et al. (30)	2007	78	21	57	33%	98%	7	1	14	56
Worthington et al. (29)	2010	46	16	30	6.25	100	1	0	15	30
Glehr et al. (28)	2013	84	55	29	80	37	44	18	11	11
Randau et al. (27)	2014	120	48	72	12.9	100	6	0	42	72
Yuan et al. (26)	2015	71	25	46	80	74	20	12	5	34
Yildirim et al. (25)	2017	80	45	40	80	60	36	16	9	24
Sa-ngasoongsong et al. (14)	2018	32	20	12	40	100	8	0	12	12
Busch et al. (24)	2020	70	23	47	13	91	3	4	20	43
Chu et al. (23)	2020	35	16	19	46	95	7	1	39	18

N-PJI, non-PJI.

### Quality and publication biases of the included studies

**Figure 2:**
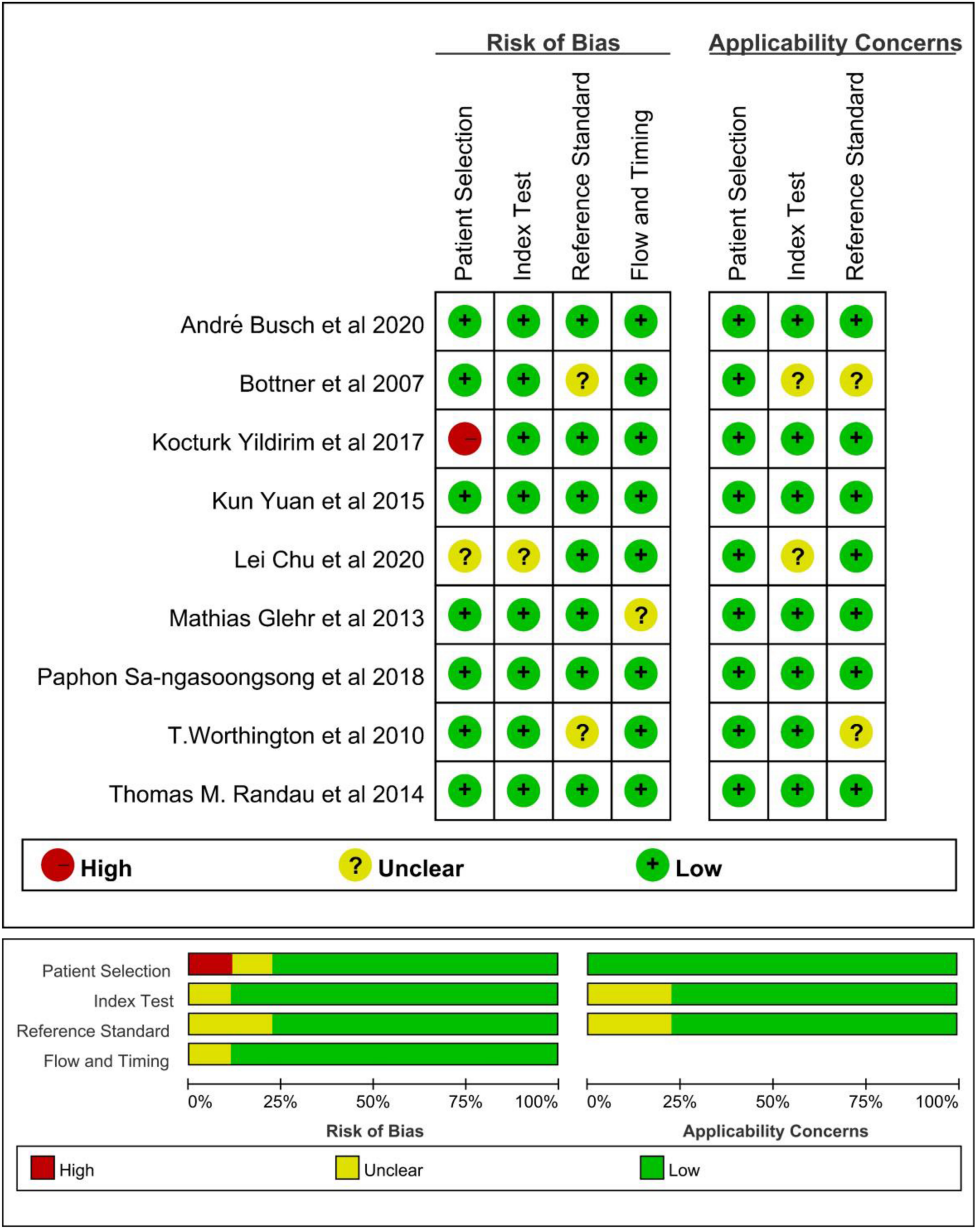
Quality assessment of included studies based on QUADAS-2 tool criteria.

**Figure 3:**
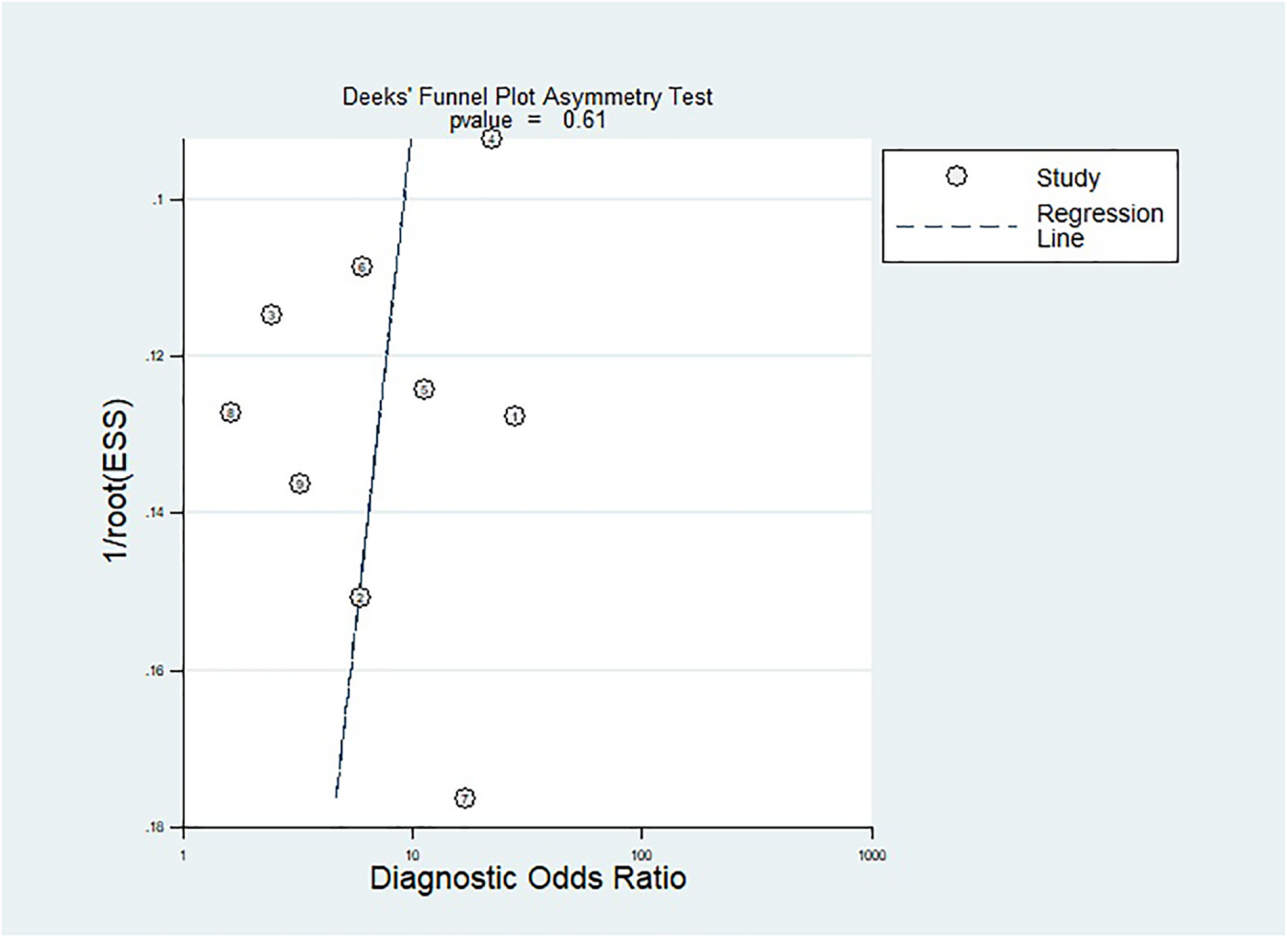
Funnel plot for publication bias assessment of included studies.

The methodological quality of the studies was assessed by using the QUADAS-2 tool (Figure 2). There is one “high risk” for patient selection, which is shown in the figure (25). Generally speaking, the quality of the included studies was satisfactory. As mentioned previously, Deeks’ test was used to analyze the publication bias of the nine included studies. The Stata command for publication bias in diagnostic meta is “midas tp fp fn tn, pubbias”. As shown in Figure 3, the funnel plot is roughly symmetric (*P* = 0.61). Therefore, there was no significant bias in publication.

### Threshold and diagnostic accuracy of PCT for PJI

The data were imported into the Meta-DiSc 1.4 software for analysis. The Spearman correlation coefficient between the logarithm of SN and 1 − SP was 0.729 (*P *= 0.026). The result was significant, indicating a threshold effect in this study. Furthermore, a “shoulder-arm” point distribution on the SROC curve graph was drawn symmetrically. It also demonstrated the threshold effects of this study. The heterogeneity might be related to the threshold effects.

The value of Cochran’s Q was 10.21 in the DOR graph (*P* = 0.250, *P* > 0.001). In other words, no heterogeneity was caused by a non-threshold effect in this study. Furthermore, the *I*^2^ statistics for DOR, SN, SP, PLR, and NLR were 21.6%, 93.9%, 92.3%, 64.2%, and 85.1%, respectively. Therefore, we used the fixed-effect model to combine the aforementioned five effects.

**Figure 4:**
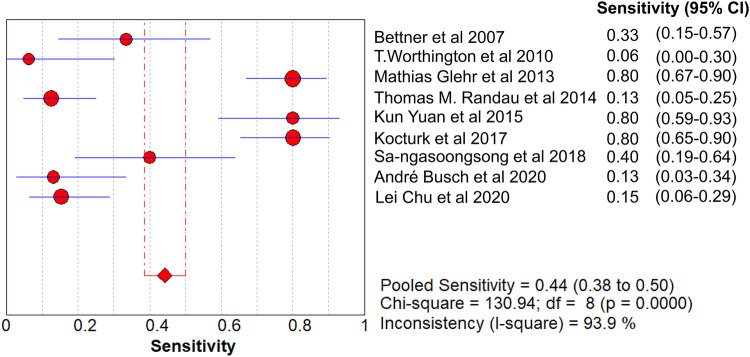
Forest plot of pooled sensitivity.

**Figure 5:**
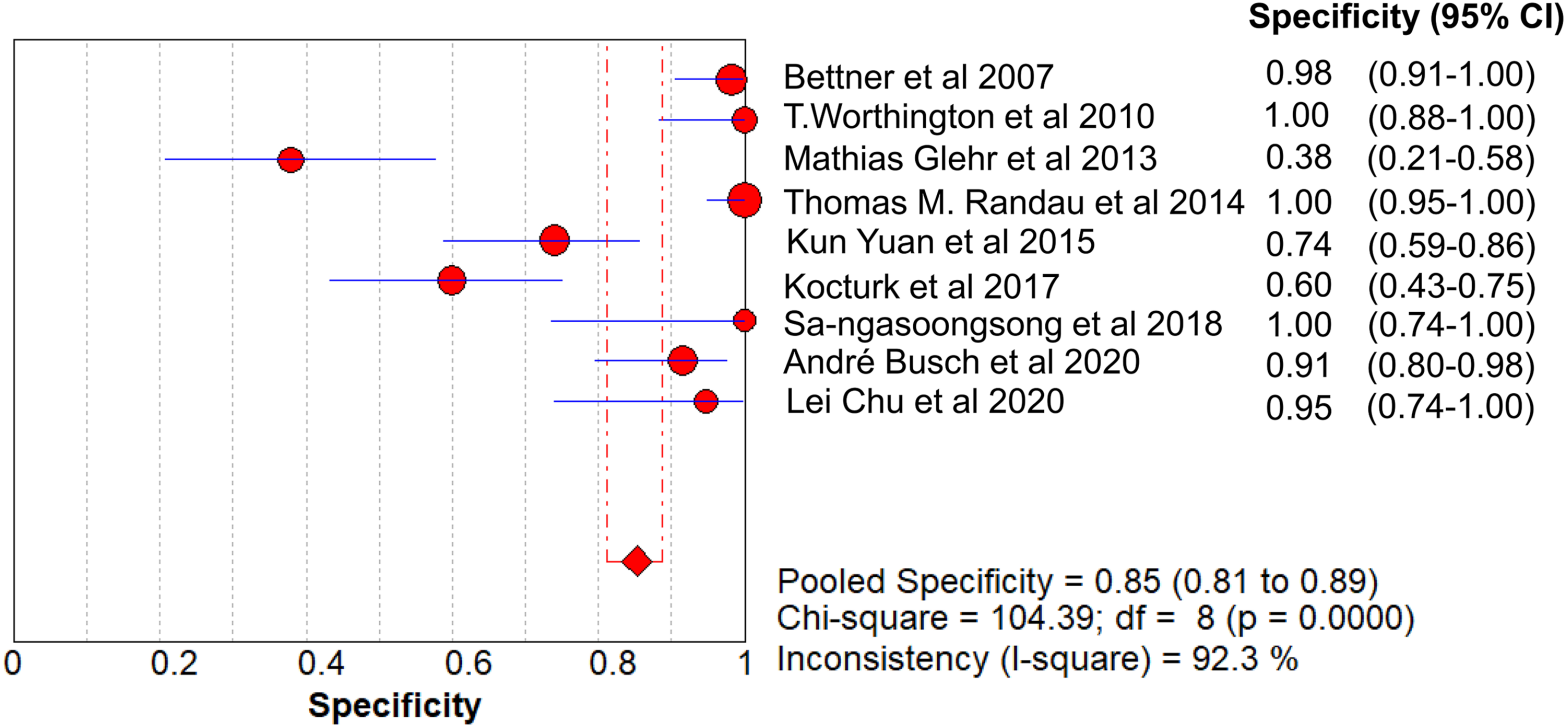
Forest plot of pooled specificity.

**Figure 6:**
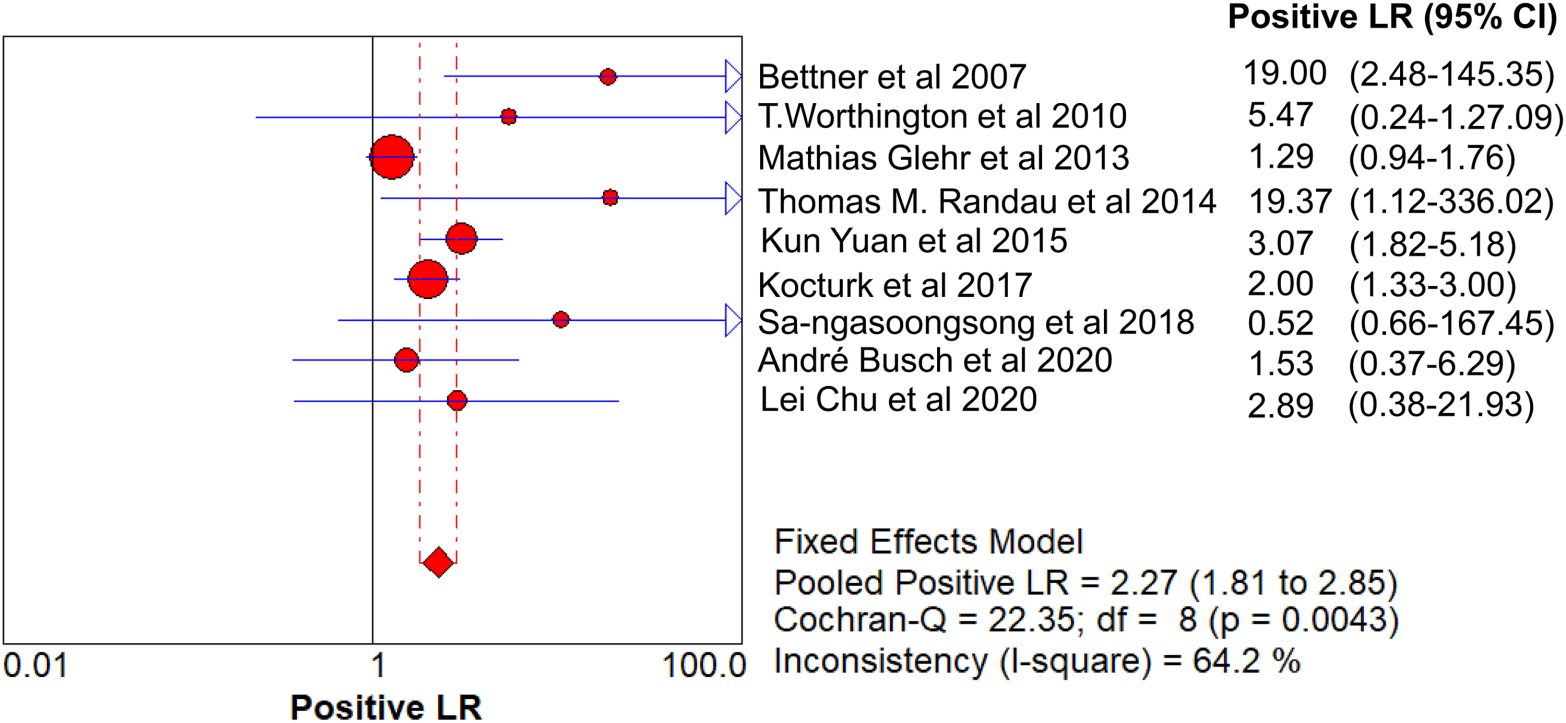
Forest plot of pooled PLR.

**Figure 7:**
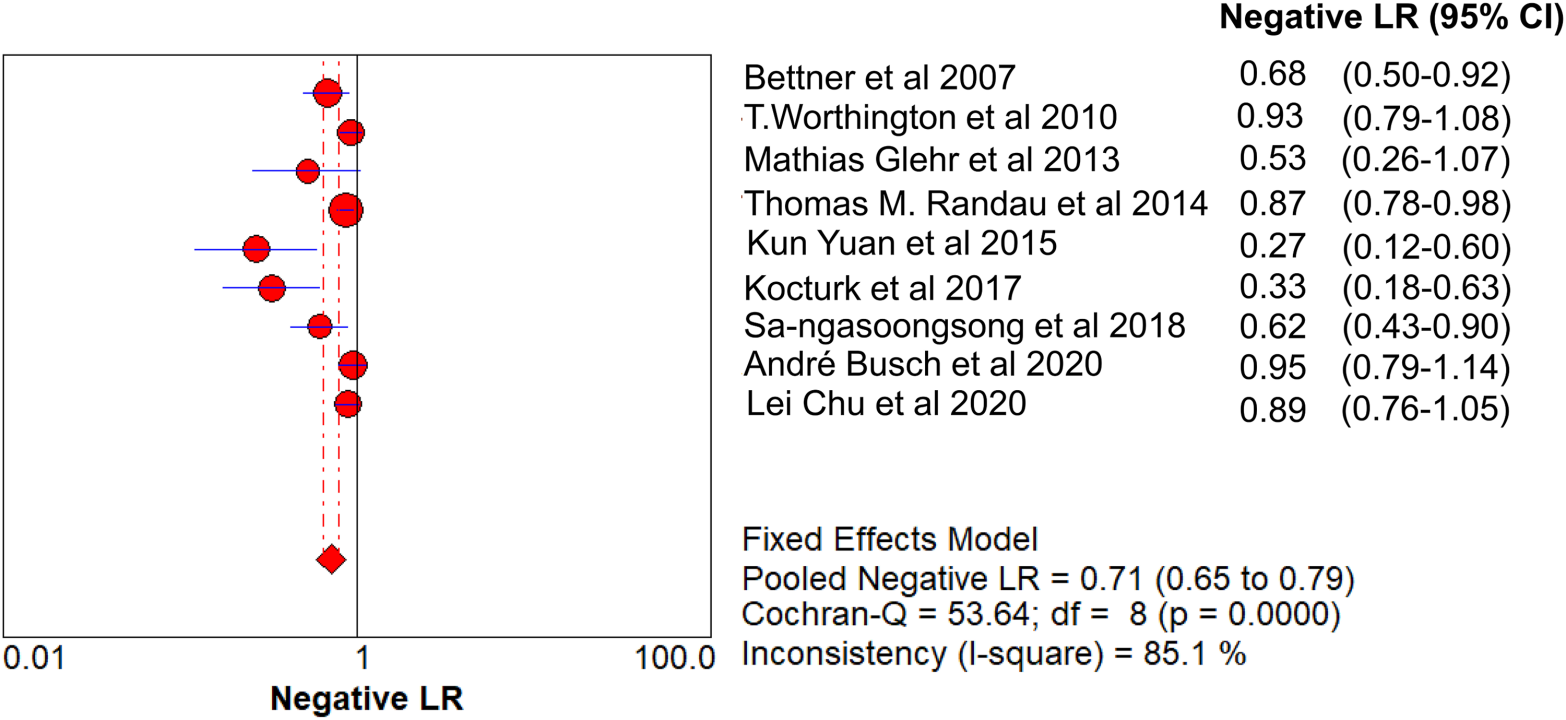
Forest plot of pooled NLR.

**Figure 8:**
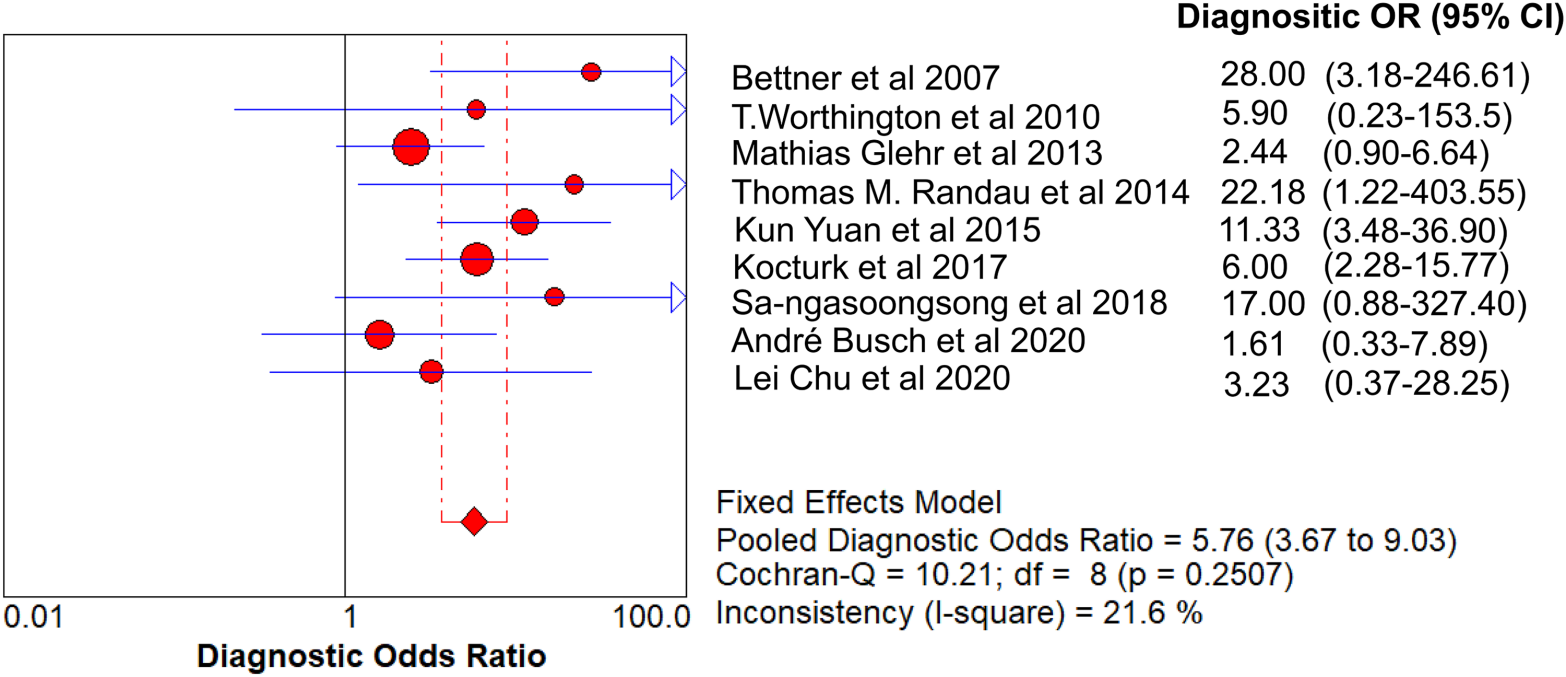
Forest plot of pooled DOR.

**Figure 9:**
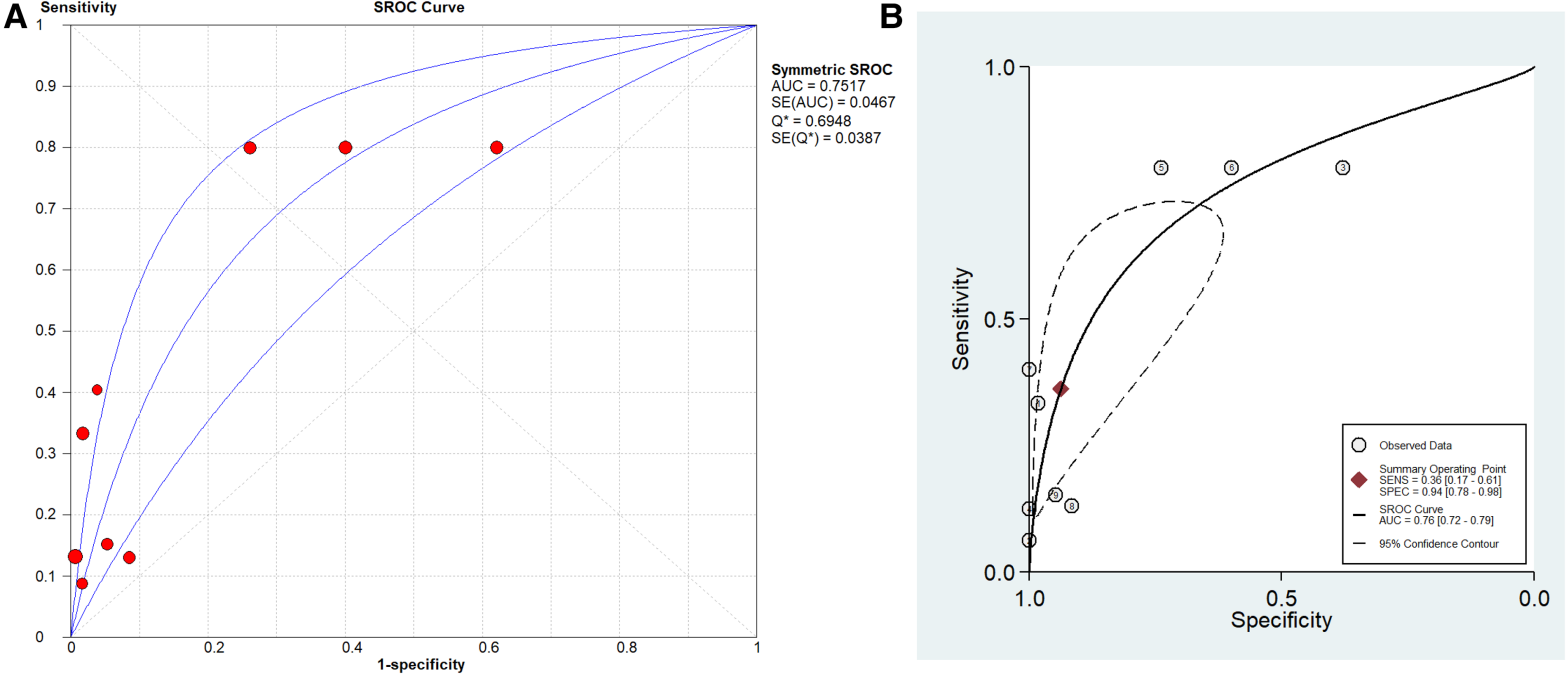
SROC curve of included studies. (**A**) Stata 14.0 software for analysis and (**B**) Meta-DiSc 1.4 software for analysis.

The pooled SN of PCT for PJI diagnosis was 0.441 [95% confidence interval (CI), 0.384–0.500] (Figure 4), the pooled SP was 0.852 (95% CI, 0.811–0.888) (Figure 5), the pooled PLR was 2.271 (95% CI, 1.808–2.853) (Figure 6), the pooled NLR was 0.713 (95% CI, 0.646–0.786) (Figure 7), and the pooled DOR was 5.756 (95% CI, 3.673–9.026) (Figure 8). The area under SROC (the pooled AUC) was 0.76 (0.72–0.79) (Figure 9A). Q index was 0.6948 (Figure 9B).

The PLR value (2.271 < 5) and NLR value (0.713 > 0.2) indicated inferior diagnostic evidence of PCT for ruling in/ruling out diagnoses. The AUC value (0.76 < 0.8) showed a low diagnostic accuracy of PCT, and the low value of DOR indicated that PCT for PJI diagnosis was an inferior target.

**Figure 10:**
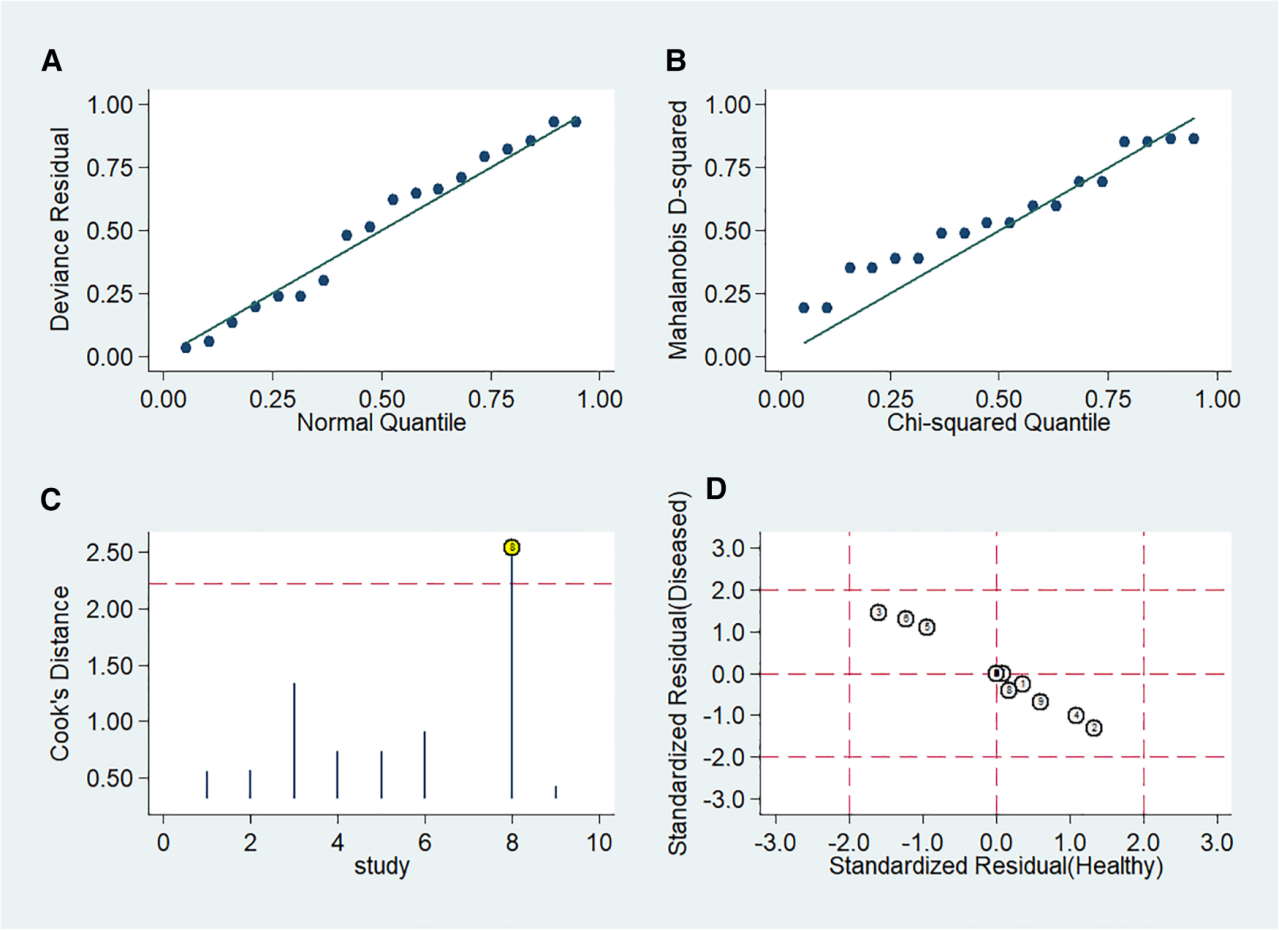
Influence of included studies. (**A**) Goodness-of-Fit. (**B**) Bivariate normality. (**C**) Influence analysis. (**D**) Outlier detection.

Stata 14.0 was selected for sensitivity analysis of the data in this study. The Stata command for sensitivity analysis of this diagnostic meta-analysis was “midas tp fp fn tn, modchk (all).” One original study (24) caused the sensitivity of results (Figure 10).

### Meta-regression and subgroup analysis

Univariate meta-regression analysis was not conducted because there was no heterogeneity caused by a non-threshold effect in this study. But we performed the following subgroup analysis for variables “Study design, Threshold value, MSIS, East Asian race” (Table 3). The subgroup analysis involves categorizing participants in a study based on certain specific features, which can help researchers explore differences in reactions among different population groups and validate the results of the main analysis.

**Table 3:** Subgroup analysis of procalcitonin for PJI diagnosis.

Subgroup	Number of studies	Sensitivity (95% CI)	Specificity (95% CI)	PLR (95% CI)	NLR (95% CI)	DOR (95% CI)
Overall studies	9	0.441 (0.384–0.500)	0.852 (0.811–0.888)	2.271 (1.808–2.853)	0.713 (0.646–0.786)	5.756 (3.673–9.026)
Study design						
Retrospective	2	0.473 (0.367–0.580)	0.712 (0.579–0.822)	2.069 (1.369–3.126)	0.614 (0.477–0.792)	5.276 (2.25–12.367)
Prospective	7	0.428 (0.360–0.498)	0.881 (0.838–0.915)	2.373 (1.805–3.120)	0.739 (0.665–0.820)	5.977 (3.494–10.224)
Threshold value						
0.5 ng/ml	4	0.381 (0.277–0.493)	0.881 (0.815–0.931)	3.185 (1.918–5.287)	0.705 (0.598–0.832)	6.643 (3.027–14.575)
Other	5	0.465 (0.397–0.534)	0.834 (0.778–0.881)	2.015 (1.564–2.595)	0.717 (0.635–0.810)	5.345 (3.062–9.332)
Diagnostic criteria						
MSIS	4	0.479 (0.407–0.552)	0.781 (0.709–0.843)	1.799 (1.390–2.328)	0.727 (0.637–0.829)	4.503 (2.505–8.093)
Race						
East Asian race	3	0.385 (0.284–0.492)	0.831 (0.729–0.907)	3.482 (2.014–6.021)	0.599 (0.476–0.754)	8.988 (3.392–23.815)

In the subgroup of retrospective studies (23, 25), the pooled SN, SP, PLR, NLR, and DOR were 0.473 (95% CI, 0.367–0.580), 0.712 (95% CI, 0.579–0.822), 2.069 (95% CI, 1.369–3.126), 0.614 (95% CI, 0.477–0.792), and 5.276 (95% CI, 2.25–12.367), respectively. In the subgroup of prospective studies (14, 24, 26–30), the pooled SN, SP, PLR, NLR, and DOR were 0.428 (95% CI, 0.360–0.498), 0.881 (95% CI, 0.838–0.915), 2.373 (95% CI, 1.805–3.120), 0.739 (95% CI, 0.665–0.820), and 5.977 (95% CI, 3.494–10.224), respectively. In the subgroup of threshold value with 0.5 ng/ml (14, 24, 26, 29), the pooled SN, SP, PLR, NLR, and DOR were 0.381 (95% CI, 0.277–0.493), 0.881 (95% CI, 0.815–0.931), 3.185 (95% CI, 1.918–5.287), 0.705 (95% CI, 0.598–0.832), and 6.643 (95% CI, 3.027–14.575), respectively. In the subgroup of “gold standard” with MSIS (23, 25, 27, 28), the pooled SN, SP, PLR, NLR, and DOR were 0.479 (95% CI, 0.407–0.552), 0.781 (95% CI, 0.709–0.843), 1.799 (95% CI, 1.390–2.328), 0.727 (95% CI, 0.637–0.829), and 4.503 (95% CI, 2.505–8.093), respectively. In the subgroup of East Asian race (14, 23, 26), the pooled SN, SP, PLR, NLR, and DOR were 0.385 (95% CI, 0.284–0.492), 0.831 (95% CI, 0.729–0.907), 3.482 (95% CI, 2.014–6.021), 0.599 (95% CI, 0.476–0.754), and 8.988 (95% CI, 3.392–23.815), respectively (Table 3).

## Discussion

Patients suffering from PJI often need long-term antibiotic treatment, secondary revision surgery, joint fusion, and amputation if infection is out of control (31). Early diagnosis of PJI is essential for orthopedic surgeons. It is possible for doctors to perform debridement, antibiotics, and retention surgery without requiring secondary revision surgery for acute PJI that occurs early after surgery (32).

It is difficult to diagnose early postoperative infection only relying on serum biomarkers (such as WBC, CRP, and ESR) as they are physiologically increased due to the healing process in the early postoperative stage (33, 34). ESR and CRP were found to be highly sensitive and lowly specific under all inflammatory conditions (35, 36). CRP often rised continuously within 30–60 days after surgery. ESR and CRP have limitations on the diagnosis of PJI (11). PCT is one of the most popular indicators in the field of infection diagnosis in recent years. It is composed of 116 amino acids and is produced by Thyroid C cells under physiological conditions. During infection, the body produces a large amount of PCT under the action of various cytokines and bacterial endotoxins. PCT is a peptide hormone of calcitonin precursors or a pro-inflammatory cytokine. Bacterial endotoxins cause elevated levels of PCT in serum. In addition, some non-bacterial infections, such as multiple organ failure and large area burn, can also lead to a significant increase in serum calcitonin (37). It is suggested in many studies that PCT is a sensitive marker of bacterial infection (38–40). Therefore, it can be used to accurately distinguish bacterial infection from non-bacterial infections (41). It is increasingly used to detect and guide the use of antibiotics (42). It has also become a sensitive and specific marker for the diagnosis of septic arthritis and acute osteomyelitis (43). Elevated PCT has high specificity for sepsis caused by bacterial infection (15). It can be used as an indicator for the diagnosis of sepsis, the identification of severe bacterial infection, and the determination of the severity of the disease. In the field of joint replacement, there was no consensus on the value of PCT in diagnosing PJI. PCT was not useful in the diagnosis of local infections such as PJI, but it is important in the diagnosis of sepsis and systemic infection (44, 45). Bouaicha et al. showed PCT was not the best indicator for the diagnosis of bone and joint infections (46). Therefore, it could be used as an indicator for inclusion diagnosis instead of exclusion diagnosis because of low sensitivity and high specificity (46).

Glehr et al. found that the serum PCT (Elecsys BRAHMS PCT) threshold was set at 0.35 ng/L with SN (80%) and SP (37%) (28), thus currently PCT could be used as an additional test for infection diagnosis. Drago et al. suggested that PCT could not be used as a diagnostic indicator for PJI (47). Yoon et al. came to the conclusion that PCT with high PLR (12.4) could be used as inclusion criteria for the diagnosis of PJI, but PCT with high NLR (0.44) was not suitable for exclusion of diagnosis (48).

PJI is primarily an advanced infection, and the incidence of early infection is less than 1% (46). PCT responds directly to bacterial endotoxin, so local joint infection does not cause PCT elevation unless the local infection becomes a systemic infection. PJI is a local low-toxicity infection that cannot produce large amounts of PCT in most cases (49). This study summarized the results of different studies on the diagnosis of PJI by PCT, which provided a reference for clinical orthopedic surgeons. The results show that the value of serum PCT in the diagnosis of PJI is extremely limited. The pooled SN of PCT is poor, whereas its SP is high. One possible explanation is that PJI is a low-toxin infection that does not have the virulence to trigger PCT release. Second, transient bacteremia is a common phenomenon in healthy patients, even after toothbrushing, which may induce low-level PCT release. In addition, PCT penetration into the synovial fluid has been rarely studied. The extent of PCT penetration into the synovial fluid may vary among patients. Therefore, caution should be exercised when considering PCT in future clinical practice to avoid potentially misleading results (50). When a patient after THA or TKA has elevated serum PCT, one cannot arbitrarily judge it as PJI and take some radical treatment. While the number of patients in our study may seem limited at 621, it is worth noting that PJI is a rare complication with a low incidence rate. As such, the inclusion of 621 cases in our analysis actually represents a substantial sample size. Moreover, our meta-analysis incorporates data from nine separate studies originating from six distinct countries, rendering our findings remarkably representative. However, there are some limitations in our research. First, there is no gold standard for PJI diagnosis. Second, many studies have shown that the biological biomarker in synovial fluid has a high diagnostic ability for PJI (51–53). We only studied serum PCT. Third, there were few included cases, and the role of PCT for PJI diagnosis could not be completely reflected. Fourth, in the included literature, some patients with acute infection were clearly excluded, and the other part was not clearly stated. Therefore, we cannot effectively represent the proportion of acute and chronic infections. Fifth, the detection method and time are not similar in all studies, which may have a particular impact on the results.

The PLR value (2.271) and NLR value (0.713) indicate inferior diagnostic evidence of PCT for ruling-in/ruling-out diagnoses. The AUC value (0.76 < 0.80) shows a low diagnostic accuracy of PCT, and the low value of DOR indicates that PCT for PJI diagnosis is an inferior target.

## Conclusion

This study showed that PCT detection of PJI has poor diagnostic accuracy. PCT is not recommended for use as a rule-out/rule-in diagnostic tool. Consequently, further studies are needed to find markers associated with PCT to improve diagnostic ability. This study examined the current understanding of PCT's diagnostic potential in PJI, providing clinicians with an exclusive reference.
